# Monocyte/High-Density Lipoprotein Ratio Is Associated with Atrial High-Rate Episodes within One Year Detected by Cardiac Implantable Electronic Devices

**DOI:** 10.21470/1678-9741-2023-0144

**Published:** 2023-10-17

**Authors:** Lishuang Ji, Le Wang, Xuecheng Song, Mei Wei, Min Li, Mingqi Zheng, Gang Liu

**Affiliations:** 1 Department of Cardiology, The First Hospital of Hebei Medical University, Shijiazhuang, Hebei, People’s Republic of China; 2 Hebei International Joint Research Center for Structural Heart Disease, Shijiazhuang, Hebei, People’s Republic of China; 3 Hebei Key Laboratory of Heart and Metabolism, Shijiazhuang, Hebei, People’s Republic of China

**Keywords:** Atrial Fibrilation, Artificial Pacemaker, Electronics, Lipids, Monocyte, Risk Factors

## Abstract

**Objective:**

To investigate the risk factors for predicting atrial high-rate episodes (AHREs) detected by cardiac implantable electronic devices (CIEDs).

**Methods:**

A total of 140 patients with CIED in our hospital from June 2013 to June 2018 were included and were followed up to observe whether they had AHREs. AHRE are defined as atrial rate ≥ 175 times/minute, lasting > 5 minutes, and reviewed by an experienced electrophysiologist with unclear clinical diagnosis. The patients fasted for 12 hours after implantation, and blood samples were collected for biochemical, lipid, and whole blood count detection. Follow-up was regular after discharge to record follow-up data of each patient and conduct statistical analysis.

**Results:**

One hundred and forty patients were implanted with dual-chamber pacemakers, their median age was 70 years old, 44.29% were male, 27 patients had AHRE within one year, and AHRE incidence rate was 19.29%. The microcytic to hypochromic (M/H) ratio was calculated for all AHRE patients and compared with the patients without AHRE; the M/H value of AHRE patients was significantly higher. Throughout the entire follow-up period, a total of 44 patients developed AHRE; when adjusted by multivariate analysis, only M/H ratio ≥ 4.5 *vs.* < 4.5 had statistical significance, and the adjusted hazard ratio value was 4.313 (1.675-11.105).

**Conclusion:**

As an indicator, M/H ratio may play an important role in the occurrence and development of atrial fibrillation and can be used as a predictor of AHRE in patients with CIED.

## INTRODUCTION

Studies around the world have shown that the prevalence and incidence rate of atrial fibrillation (AF) are gradually increasing, which will lead to an increased mortality. The incidence rate of AF varied greatly in different regions, even varying 12-fold between regions, and was higher in North America, Europe, China, and Southeast Asia^[1,2]^. Research shows that the risk of stroke in AF patients is five-fold higher than in normal people, and the mortality rate increases by two-fold^[3]^, which requires prompt diagnosis and intervention to improve this dilemma. However, it is well established that there is a poor correlation between symptoms and AF^[4]^. Cardiac implantable electronic devices (CIEDs) are currently recognized as commonly used methods for the treatment of arrhythmias, which can be used to detect, analyze, and store atrial high-rate episodes (AHREs). This method is significantly superior to previous conventional diagnostic methods, such as resting electrocardiogram and Holter monitoring^[5,6]^. AHREs, also referred to as “subclinical AF” or “silent AF”, are closely linked to AF without doubt^[7]^. Other studies believe that silent AF is a precursor type of clinical AF, which can significantly increase the probability of thromboembolism and even death^[8,9,10,11]^. Therefore, the early detection and early treatment of AHREs is of great clinical significance. A recent consensus from the European Heart Rhythm Association (or EHRA) suggested that clinicians should perform stroke risk stratification as well as treatment in patients with subclinical AF using the Congestive heart failure, Hypertension, Age ≥ 75 (doubled), Diabetes, Stroke (doubled), VAScular disease, age 65 to 74, and sex category (female) (CHA2DS2-VASc) score^[12]^. Thus, the aim of our study was to specifically investigate the risk factors of AHREs in patients who had undergone CIED implantation during follow-up.

## METHODS

### Patients

Retrospective analysis was made on 140 patients with dual-chamber pacemakers implanted in the First Hospital of Hebei Medical University from June 2013 to June 2018. In all patients, the attending doctor decided which device manufacturer to choose. This study protocol was approved by the Ethics Committee of the First Affiliated Hospital of Hebei Medical University (20200369), and informed consent was obtained from all the study subjects before enrollment. Patients with renal failure, heart valve disease, atrial arrhythmia, history of valvoplasty or valve replacement surgery, and pacemaker installation were excluded.

### Inclusion and Exclusion Criteria

Inclusion criterion was patients with a dual-chamber pacemaker (automatic mode switch [AMS] function) for bradycardia (including sick sinus node syndrome or atrioventricular block). And exclusion criteria were (1) patients implanted with single-chamber pacemakers (VVI and AAI devices) or changed from DDD pacemaker to VVI and AAI modes; (2) previous preoperative history of rapid atrial arrhythmia (including atrial tachycardia, atrial flutter, and AF); (3) < 18 years old; (4) left atrial internal diameter > 65 mm; (5) previous history of congenital heart disease, internal interventional cardiac valvuloplasty or valve replacement, history of cardiac surgery or having thyroid dysfunction, and severe cardio-renal insufficiency; (6) follow-up time < 12 months; (7) AMS function not turned on at one week after implantation; and (8) incomplete medical records and follow-up data.

### Observation Indicator

We recorded and summarized general information (including demographic characteristics) and relevant clinical information of all patients. Each patient was implanted with a dual-chamber pacemaker, which was programmed into dual-chamber rate-modulated (or DDDR) mode and kept in the atrial tachycardia detection mode, so as to inhibit AF by atrial overdrive pacing. Properly inquire about the sensitivity of bipolar atrial leads and post-ventricular atrial blanking period to reduce P wave sensitivity and far field R wave hypersensitivity was done to identify atrial activity during AHRE. AHREs refer to AF > 175 bpm and lasting > 5 minutes. After fasting for 12 hours, blood samples were collected for biochemical, lipid, and whole blood count tests. The reference value for monocyte count in our laboratory was 2-10% of the total white blood cell count.

### Follow-up

Patients were followed up in the hospital for one, three, and six months and one year after discharge; then, they were followed up once a year. Follow-up data of each patient were recorded and registered (including 12-lead electrocardiogram, pacemaker program control data, test results, etc.). The softwares of American Medtronic company and St. Jude company were used to regularly conduct routine control analysis of their respective brand pacemakers.

### Statistical Analysis

Quantitative variables were converted to dichotomous variables according to their mean or median. Categorical variables were expressed as frequency. All patients were followed up for one full year, and chi-square test or Fisher’s exact test was used to compare the AHRE rates of patients with different characteristics within one year after surgery. The Kaplan-Meier method was used to draw the survival curve of patients with AHRE after implantation, and the log-rank method was used to compare the curves. Cox model was used to analyze the risk factors of AHRE occurrence, and hazard ratio (HR) values were calculated. Statistical significance was set at < 0.05. All tests were two-tailed, and analysis was carried out using statistical analysis software (SAS 9.3).

## RESULTS

### General Characteristics

A total of 140 patients with CIED implantation were chosen for postoperative follow-up and observation, the average length of follow-up was 39.26±24.18 months (range: 12 to 102 months), the median age was 70 years (interquartile range: 61-75), and 44.29% of them were males. The preoperative mean CHA2DS2-VASc score was 2.94±1.81. Main surgery causes were sick sinus (52.86%) and atrioventricular block (42.86%).

### AHRE Analysis Occurring in One Year

AHRE occurred in a total of 27 cases within one year, with an incidence of 19.29%. The occurrence of one-year AHRE of patients with different age, gender, body mass index, surgery cause, CHA_2_DS_2_-VASC score, and previous history were listed in Table 1. The incidence of male patients was significantly higher than that of female patients (27.4% *vs.* 12.82%, respectively; *P*=0.03).

**Table 1:** Clinical characteristics of patients according to the occurrence of AHREs during one-year follow-up.

Variables	Level	No	Yes	Total	Test method	Statistics	P-value
Gender	Male	45 (72.58)	17 (27.42)	62 (44.29)	Chi-square test	4.729	0.030
	Female	68 (87.18)	10 (12.82)	78 (55.71)			
Age	1 ≥ 70	57 (80.28)	14 (19.72)	71 (50.71)	Chi-square test	0.017	0.895
	2 < 70	56 (81.16)	13 (18.84)	69 (49.29)			
BMI	1 ≥ 25	50 (81.97)	11 (18.03)	61 (43.57)	Chi-square test	0.109	0.741
	2 < 25	63 (79.75)	16 (20.25)	79 (56.43)			
CHA_2_DS_2_-VASc score	1 ≥ 3	65 (81.25)	15 (18.75)	80 (57.14)	Chi-square test	0.034	0.853
	2 < 3	48 (80.00)	12 (20.00)	60 (42.86)			
Disease type	Sick sinus	60 (81.08)	14 (18.92)	74 (52.86)	Fisher’s exact test	-	0.661
	Atrioventricular block	49 (81.67)	11 (18.33)	60 (42.86)			
	Other	4 (66.67)	2 (33.33)	6 (4.29)			
Smoking	No	100 (81.30)	23 (18.70)	123 (87.86)	Fisher’s exact test	-	0.743
	Yes	13 (76.47)	4 (23.53)	17 (12.14)			
Drinking	No	99 (80.49)	24 (19.51)	123 (87.86)	Fisher’s exact test	-	1.000
	Yes	14 (82.35)	3 (17.65)	17 (12.14)			
Hypertension	No	41 (74.55)	14 (25.45)	55 (39.29)	Chi-square test	2.215	0.137
	Yes	72 (84.71)	13 (15.29)	85 (60.71)			
CHD	No	65 (81.25)	15 (18.75)	80 (57.14)	Chi-square test	0.034	0.853
	Yes	48 (80.00)	12 (20.00)	60 (42.86)			
Diabetes	No	94 (81.74)	21 (18.26)	115 (82.14)	Fisher’s exact test	-	0.577
	Yes	19 (76.00)	6 (24.00)	25 (17.86)			
Heart failure	No	97 (82.20)	21 (17.80)	118 (84.29)	Fisher’s exact test	-	0.376
	Yes	16 (72.73)	6 (27.27)	22 (15.71)			
Hyperlipidemia	No	98 (80.33)	24 (19.67)	122 (87.14)	Fisher’s exact test	-	1.000
	Yes	15 (83.33)	3 (16.67)	18 (12.86)			

AHREs=atrial high-rate episodes; BMI=body mass index; CHA_2_DS_2_-VASc=Congestive heart failure, Hypertension, Age ≥ 75 (doubled), Diabetes, Stroke (doubled), VAScular disease, age 65 to 74, and sex category (female); CHD=chronic heart disease

The characteristics of patients whether AHRE occurred or not within one year after implantation according to patients’ cardiac indexes, implantation site, and biochemical indexes were listed in Table 2. The results demonstrated that patients with left ventricular end-diastolic diameter ≥ 50 mm, right atrial transverse diameter (RATD) ≥ 36 mm, and left ventricular volume ≥ 120 had a higher incidence. Moreover, there were significant differences in the incidence of AHRE among various levels of neutrophils, monocytes, and lipoprotein A and microcytic to hypochromic (M/H) value. Of which, the comparison result of M/H ratio ≥ 4.5 *vs.* < 4.5 had the greatest difference (37.70% *vs.* 5.06%, respectively; *P*<0.001).

**Table 2:** Incidence of AHRE within one year after implantation according to the implantation site and cardiac indexes.

Variables	Level	No	Yes	Total	Test method	Statistics	P-value
Work mode	DDD	109 (81.34)	25 (18.66)	134 (95.71)	Fisher’s exact test	-	0.327
	Other	4 (66.67)	2 (33.33)	6 (4.29)			
Implantation site	High and low interval	12 (70.59)	5 (29.41)	17 (12.14)	Fisher’s exact test	-	0.498
	Right ventricular apex	84 (81.55)	19 (18.45)	103 (73.57)			
	Median septum	17 (85.00)	3 (15.00)	20 (14.29)			
Atrial pacing ratio	1 ≥ 0.42	48 (81.36)	11 (18.64)	59 (42.14)	Chi-square test	0.027	0.870
	2 < 0.42	65 (80.25)	16 (19.75)	81 (57.86)			
Ventricular pacing ratio	1 ≥ 0.48	53 (79.10)	14 (20.90)	67 (47.86)	Chi-square test	0.214	0.644
	2 < 0.48	60 (82.19)	13 (17.81)	73 (52.14)			
Preoperative heart rate	1 ≥ 60	44 (81.48)	10 (18.52)	54 (38.57)	Chi-square test	0.033	0.855
	2 < 60	69 (80.23)	17 (19.77)	86 (61.43)			
LVEDD	1 ≥ 50	48 (71.64)	19 (28.36)	67 (47.86)	Chi-square test	6.794	0.009
	2 < 50	65 (89.04)	8 (10.96)	73 (52.14)			
RATD	1 ≥ 36	42 (72.41)	16 (27.59)	58 (41.43)	Chi-square test	4.383	0.036
	2 < 36	71 (86.59)	11 (13.41)	82 (58.57)			
Left ventricular volume	1 ≥ 120	39 (69.64)	17 (30.36)	56 (40.00)	Chi-square test	7.350	0.007
	2 < 120	74 (88.10)	10 (11.90)	84 (60.00)			
Ejection fraction	1 ≥ 65	67 (81.71)	15 (18.29)	82 (58.57)	Chi-square test	0.125	0.723
	2 < 65	46 (79.31)	12 (20.69)	58 (41.43)			

AHRE=atrial high-rate episode; DDD=; LVEDD=left ventricular end-diastolic diameter; RATD=right atrial transverse diameter

The RR values of the abovementioned variables with significances in the occurrence of AHRE within one year were displayed in Table 3.

**Table 3:** Comparison of patients with or without AHRE within one year after implantation according to preoperative biochemical indexes.

Variable	Level	No	Yes	Total	Test method	Statistics	P-value
Neutrophils	1 ≥ 4.3	38 (69.09)	17 (30.91)	55 (39.29)	Chi-square test	7.862	0.005
	2 < 4.3	75 (88.24)	10 (11.76)	85 (60.71)			
Lymphocyte	1 ≥ 1.9	39 (75.00)	13 (25.00)	52 (37.14)	Chi-square test	1.735	0.188
	2 < 1.9	74 (84.09)	14 (15.91)	88 (62.86)			
Monocyte	1 ≥ 0.48	48 (68.57)	22 (31.43)	70 (50.00)	Chi-square test	13.261	0.000
	2 < 0.48	65 (92.86)	5 (7.14)	70 (50.00)			
Platelet	1 ≥ 190	49 (80.33)	12 (19.67)	61 (43.57)	Chi-square test	0.010	0.919
	2 < 190	64 (81.01)	15 (18.99)	79 (56.43)			
Creatinine	1 ≥ 88	26 (72.22)	10 (27.78)	36 (25.71)	Chi-square test	2.245	0.134
	2 < 88	87 (83.65)	17 (16.35)	104 (74.29)			
Uric acid	1 ≥ 350	46 (77.97)	13 (22.03)	59 (42.14)	Chi-square test	0.495	0.482
	2 < 350	67 (82.72)	14 (17.28)	81 (57.86)			
Fasting plasma glucose	1 ≥ 5.5	41 (80.39)	10 (19.61)	51 (36.43)	Chi-square test	0.005	0.942
	2 < 5.5	72 (80.90)	17 (19.10)	89 (63.57)			
High-density lipoprotein	1 ≥ 1.0	77 (85.56)	13 (14.44)	90 (64.29)	Chi-square test	3.794	0.051
	2 < 1.0	36 (72.00)	14 (28.00)	50 (35.71)			
Low-density lipoprotein	1 ≥ 3	47 (82.46)	10 (17.54)	57 (40.71)	Chi-square test	0.187	0.665
	2 < 3	66 (79.52)	17 (20.48)	83 (59.29)			
Apolipoprotein A1	1 ≥ 1.3	48 (84.21)	9 (15.79)	57 (40.71)	Chi-square test	0.755	0.385
	2 < 1.3	65 (78.31)	18 (21.69)	83 (59.29)			
Apolipoprotein B	1 ≥ 0.86	58 (80.56)	14 (19.44)	72 (51.43)	Chi-square test	0.002	0.961
	2 < 0.86	55 (80.88)	13 (19.12)	68 (48.57)			
Apolipoprotein A	1 ≥ 250	30 (69.77)	13 (30.23)	43 (30.71)	Chi-square test	4.778	0.029
	2 < 250	83 (85.57)	14 (14.43)	97 (69.29)			
Alanine aminotransferase	1 ≥ 25	33 (82.50)	7 (17.50)	40 (28.57)	Chi-square test	0.115	0.735
	2 < 25	80 (80.00)	20 (20.00)	100 (71.43)			
M/H ratio	1 ≥ 4.5	38 (62.30)	23 (37.70)	61 (43.57)	Chi-square test	23.561	0.000
	2 < 4.5	75 (94.94)	4 (5.06)	79 (56.43)			

AHRE=atrial high-rate episode; M/H=microcytic to hypochromic

After adjusted by the generalized linear model multivariate analysis, only M/H ratio ≥ 4.5 *vs.* < 4.5 had statistical significance, and the adjusted RR value was 5.95 (2.334-17.23), which was displayed in Table 4.

**Table 4:** Risk factors for AHRE within one year after implantation.

Risk factor	Level	Single-factor analysis		Multi-factor analysis*	
		RR (95% CI)	P-value	RR (95% CI)	P-value
Gender	Male *vs.* Female	2.139 (1.055-4.334)	0.030	0.858 (0.82-1.897)	0.689
LVEDD	≥ 50 *vs. <* 50	2.588 (1.214-5.5143)	0.009	2.093 (0.696-3.649)	0.177
RATD	≥ 36 *vs. <* 36	2.056 (1.031-4.100)	0.036	1.45 (0.775-3.147)	0.320
LV	≥ 120 *vs. <* 120	2.550 (1.261-5.156)	0.007	0.979 (0.539-2.848)	0.968
Neutrophils	≥ 4.3 *vs. <* 4.3	2.627 (1.300-5.310)	0.005	1.593 (0.903-3.228)	0.145
Apolipoprotein A	≥ 250 *vs. <* 250	2.095 (1.078-4.069)	< 0.001	1.523 (0.829-1.715)	0.200
M/H ratio	≥ 4.5 *vs. <* 4.5	7.447 (2.718-20.402)	< 0.001	5.95 (2.334-17.23)	0.001

AHRE=atrial high-rate episode; CI=confidence interval; LV=left ventricle; LVEDD=left ventricular end-diastolic diameter; M/H=microcytic to hypochromic; RATD=right atrial transverse diameter; RR=risk ratio

* Multi-factor analysis was done using generalized linear model

**Fig. 1:**
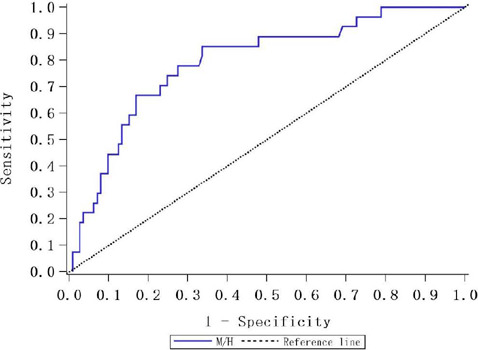
Receiver operating characteristic curve of microcytic to hypochromic (M/H) ratio in predicting atrial high-rate episodes within one year.

The receiver operating characteristic curve of M/H ratio in predicting AHRE within one year was presented in Figure 1, the area under the curve was 0.793 (95% confidence interval, 0.699-0.887). The cutoff value was 4.5 when Youden Index reached maximum, and the sensitivity and specificity were 0.852 and 0.664, respectively.

### Time Survival Analysis of AHRE After Implantation

**Fig. 2:**
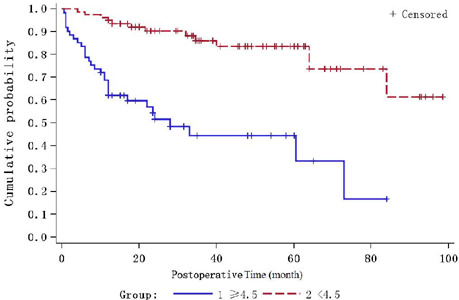
- Probability curve of atrial high-rate episodes during follow-up.

Throughout the entire follow-up period, a total of 44 patients developed AHRE. Kaplan-Meier method was used to draw the time probability curve of AHRE in patients with different demographic, clinical, and biochemical characteristics after CIED implantation. There were statistical differences only in patients with different right atrial diameter, left ventricular end-diastolic diameter, neutrophil count, lymphocyte count, monocyte count, and M/H level (*P*<0.05). The HR values of the abovementioned six variable risk factors calculated by the Cox model were exhibited in Table 5. When adjusted by multivariate analysis, only M/H ratio ≥ 4.5 *vs.* < 4.5 had statistical significance, and the adjusted HR value was 4.313 (1.675-11.105). The probability curve of AHRE during follow-up was demonstrated in Figure 2.

**Table 5:** Risk factors for AHRE after implantation (Cox analysis).

Risk factor	Level	Single-factor analysis		Multi-factor analysis	
		HR (95% CI)	P-value	HR (95% CI)	P-value
RATD	≥ 36 *vs. <* 36	2.167 (1.182-3.972)	0.012	1.754 (0.899-3.422)	0.099
LV	≥ 120 *vs. <* 120	1.857 (1.022-3.374)	0.042	1.44 (0.729-2.847)	0.294
Neutrophils	≥ 4.3 *vs.* < 4.3	2.081 (1.142-3.79)	0.017	1.572 (0.784-3.149)	0.202
Lymphocyte	≥ 1.9 *vs. <* 1.9	2.146 (1.184-3.89)	0.012	1.352 (0.715-2.556)	0.353
Monocyte	≥ 0.48 *vs. <* 0.48	3.418 (1.774-6.586)	< 0.001	0.882 (0.315-2.469)	0.812
M/H ratio	≥ 4.5 *vs.* < 4.5	5.163 (2.663-10.009)	< 0.001	4.313 (1.675-11.105)	0.003

AHRE=atrial high-rate episode; CI=confidence interval; HR=hazard ratio; LV=left ventricle; RATD=right atrial transverse diameter

## DISCUSSION

The main purpose of this study was to explore the risk factors that affect the occurrence of AHRE in patients with CIEDs. After the follow-up of 140 patients with AHRE, we found that the probability of AHRE in patients with high M/H ratio was significantly higher and predicted more adverse outcome. Previous studies had shown that aging, hypertension, diabetes, heart failure, cardiovascular disease, etc. were closely related to the occurrence and development of AF. However, there was no conclusive evidence that these factors can predict the occurrence of AHRE^[9,13]^.

Jelavic and Tse’s research concludes that there is gender difference in cardiac electrophysiology, and shorter atrial effective refractory period (ERP) can promote electrical remodeling of AF. It was found that the ERP of premenopausal women is shorter than that of postmenopausal women and men^[2,14]^. Thus, female sex hormones may be protective against AF, and consistent with this, we found that the incidence of AHRE of females was lower than that of males in the univariate analysis although there was no difference in multivariate analysis.

Among the patients’ cardiac and biochemical indexes, we found some factors which might affect the development of AHRE, including left ventricular end-diastolic diameter, RATD, left ventricular volume, levels of neutrophils, monocytes, and lipoprotein A, and M/H value. Pastori and Li et al.^[15,16]^ showed that some previous clinical history and laboratory test results were closely related to the occurrence of AHRE. In addition, Pastori et al.^[15]^ conducted a real-world court study in patients with CIEDs, the results showed that the patient’s age, past history of AF, blood routine leukocyte count, and C-reactive protein content were closely related to AHREs. However, in the current study, multivariate analysis revealed that most factors were not related to the occurrence of AHREs, except M/H ratio.

Previous studies have shown that chronic diseases such as cardiovascular diseases, hypertension, and diabetes are accompanied by inflammation. Similarly, recent studies have also confirmed that the occurrence and development of AF are accompanied by inflammation^[12,13]^. In addition, Lamm’s article concludes that there was a significant relationship between the increased risk of postoperative AF and the increase of white blood cells^[17]^, which also confirmed the relationship between inflammation and AF to a certain extent, and may be the common pathogenic pathway of AHREs and AF^[18]^. In this process, the activation of leukocytes will produce a large number of inflammatory mediators (including cytokines, active oxidants, etc.), which can affect the myocardial tissue, leading to the occurrence and development of electrical remodeling and fibrosis^[19]^. Therefore, AHREs may be an early sign of this proinflammatory process.

M/H ratio is the ratio of monocytes to high-density lipoprotein cholesterol. In Rogacev’s research, M/H ratio is considered as a potential marker that can predict cardiovascular events^[20]^. In addition, Saskin et al.^[21]^ observed that the increase of M/H ratio was an independent risk factor for early death and postoperative AF. Çanpolat et al.^[22]^ also believed that the increase of M/H ratio was significantly positively related to the recurrence of AF after successful cryoballoon-based catheter ablation.

Silent AF refers to AF without clinical symptoms. With the development of CIED technology, it is gradually known to people. Research suggests that the first symptom of AHREs may be stroke^[23]^. A prospective study by Satilmis showed that M/H ratio was significantly increased when AHREs occurred in patients with CIEDs^[24]^. Consistent with the abovementioned study, our study proved the association with AHREs as well, with the cutoff value of ≥ 4.5, and the high M/H ratio predicted higher mortality.

### Limitations

This study still has some limitations. Our study is a single-center retrospective study, with a small sample size. In the future, we still need more samples, more centers, and even a prospective randomized controlled study to get more accurate results.

## CONCLUSION

To sum up, M/H ratio can significantly measure inflammation and oxidative stress, which may play an important role in the occurrence and development of AF, and this increased ratio can be used as a predictor of AHREs in patients with CIEDs. However, the pathogenesis of AHRE still needs further study.

## Abbreviations, Acronyms & Symbols

AF: Atrial fibrillation
AHREs: Atrial high-rate episodes
AMS: Automatic mode switch
BMI: Body mass index
CHA_2_DS_2_-VASc: Congestive heart failure, Hypertension, Age ≥ 75 (doubled), Diabetes, Stroke (doubled), VAScular disease, age 65 to 74, and sex category (female)
CHD: Chronic heart disease
CI: Confidence interval
CIEDs: Cardiac implantable electronic devices
ERP: Effective refractory period
HR: Hazard ratio
LV: Left ventricle
LVEDD: Left ventricular end-diastolic diameter
M/H: Microcytic to hypochromic
RATD: Right atrial transverse diameter
RR: Risk ratio
